# *Staphylococcus aureus* Is the Predominant Pathogen in Hospitalised Patients with Diabetes-Related Foot Infections: An Australian Perspective

**DOI:** 10.3390/antibiotics13070594

**Published:** 2024-06-26

**Authors:** Kate E. Morton, Sarah H. Coghill

**Affiliations:** 1 Pharmacy Department, Lismore Base Hospital, Lismore, NSW 2480, Australia; 2 Infectious Diseases Department, Lismore Base Hospital, Lismore, NSW 2480, Australia

**Keywords:** diabetic foot, foot ulcer, microbiology, infection

## Abstract

Diabetes prevalence continues to increase worldwide, which has led to a rising incidence of diabetes-related foot infections (DFIs). There is significant local variation in the microbiology of DFIs, and *Pseudomonas* spp. is suggested to be more prevalent in subtropical climates. The aim of this study was to investigate the local microbiological findings in patients admitted to the hospital with DFIs. This retrospective study analysed data from all adult patients diagnosed with diabetes and admitted to the hospital for the treatment of a DFI between 1 January 2021 and 31 December 2022. Both superficial wound swabs and tissue cultures were included. The Infectious Diseases Society of America classification system was used to categorise the severity of the DFI. Patient characteristics and demographics were analysed using descriptive statistics. One hundred fifty-one episodes of care were included. Most of the DFIs were classified as moderate infections 101/151 (67%). The most commonly isolated microorganism was *Staphylococcus aureus* (33%) followed by normal skin flora (11%) and β-haemolytic streptococci (7%). *P. aeruginosa* was isolated more commonly in those with chronic DFIs (10%) compared to those with acute DFIs (2%). Despite the frequent identification of *S. aureus*, 83% of patients received an antipseudomonal antibiotic. The introduction of multidisciplinary DFI rounds should be considered.

## 1. Introduction

It is projected that by 2045, approximately 1 in 10 individuals worldwide will be impacted by diabetes [1]. The increasing prevalence of diabetes mellitus globally has led to a rise in diabetes-related foot disease (DFD), including foot infections [1]. In Australia, DFD results in 28,000 hospitalisations and 5000 amputations annually [2]. A diabetes-related foot infection (DFI) is defined as the presence of an infection in any tissue below the malleolus in an individual with diabetes mellitus [1]. While these infections may initially be superficial, they can progress and become complicated by osteomyelitis (OM) [3]. DFIs can have a significant impact on patients’ mobility, independence, and overall quality of life [4]. DFIs are particularly concerning because they are the most common precipitating events leading to lower extremity amputations [5]. Lower limb amputations can be classified as minor or major and are often feared more than death by many patients [4]. The implementation of a multidisciplinary DFI team has been proven effective in reducing the need for major amputations [6].

Most DFIs are polymicrobial [4], but a recent systematic review acknowledged that there is significant local variation in the microbiology of diabetic foot infections [3]. The most frequently identified organism was *Staphylococcus aureus*, followed by *Pseudomonas* spp. and *Escherichia coli* [3]. It was identified that the prevalence of gram-positive organisms was greater among high-income countries (HICs), whilst gram-negative isolates were more prevalent among upper- and lower-middle-income countries (U/LMICs). The majority of the studies included in the review were conducted in U/LMIC countries, with a notable focus on India [3]. There was a lack of studies conducted within HICs such as Australia [3].

The International Working Group on the Diabetic Foot (IWGDF) and the Infectious Diseases Society of America (IDSA) guidelines suggest that *Pseudomonas aeruginosa* is more prevalent in (sub)tropical climates [5]. They recommend antipseudomonal therapy for moderate or severe DFIs in those with macerated wounds or in warm climates [5]. The study facility is located in a subtropical climate (northern east coast of New South Wales, Australia) [7]. The Australian Therapeutic Guidelines do not consider the climate when recommending empiric antibiotics [8]. This is despite a study in the top end of Australia demonstrating a high incidence of *P. aeruginosa* DFIs (26.6%) [9]. Antipseudomonal antibiotics are only recommended in severe infections or if there has been recent colonisation or infection with *P. aeruginosa* [8].

The IWGDF/IDSA guidelines suggest that when treating DFIs, it is important to consider the principles of antimicrobial stewardship (AMS). This is crucial due to the complex and polymicrobial nature of DFIs [5]. DFIs are often chronic and recurrent, which places patients at an increased risk for antimicrobial adverse effects such as *Clostridioides difficile* infection [10]. AMS programs should prioritise implementing strategies to optimise DFI management [10] despite antimicrobial therapy being just one component of comprehensive DFI care [4].

Given the location of the study site and the conflicting recommendations between Australian and international guidelines, a microbiology study is needed. The aim of this study was to investigate the local antimicrobial use and microbiological findings in patients with DFI at a regional centre located in a subtropical environment. The study hypothesis was that the use of antipseudomonal antibiotics was not warranted according to the local microbiology results.

## 2. Results

A total of 107 patients with 151 episodes of care were included for analysis. Twenty-four patients had >1 admission to the hospital during the two-year period. Patient demographics are outlined in Table 1. The median age was 69 years (interquartile range [IQR] 58–74), and the median BMI was 29.42 (IQR 19.28–63.20). The majority were men (104 (69%)), had type II diabetes (92%), and were assessed as having moderate-severity infections (67%). Overall, 68 (45%) episodes of care were assessed as acute infections, and 70 (46%) were diagnosed with OM. The majority of the culture results were polymicrobial (58%). In addition, 97 patients (64%) received antibiotics prior to admission, and 127 (84%) received an antipseudomonal antibiotic during the hospital admission. The median duration of antibiotics was 15 days (IQR 11–20). The median duration of antibiotics post-amputation was 10 days (IQR 7–13 days).

A total of 256 microorganisms were identified through superficial and tissue samples. The frequency of the microorganisms can be seen in Table 2. *S. aureus* was the most commonly identified microorganism in acute DFIs, followed by normal skin flora and β-haemolytic streptococci. *S. aureus* was the most commonly identified microorganism in chronic DFIs, followed by *P. aeruginosa* and normal skin flora. *S. aureus* was the most commonly isolated microorganism, followed by normal skin flora and β-haemolytic streptococci out of all superficial and tissue samples, which included acute, chronic, and non-assessable infections. The majority of the microorganisms from all superficial and tissue samples (n = 256) were gram-positive (n = 128; 50%), whilst 26% (n = 67) were gram-negative. Other organisms accounted for the remaining 24% (n = 61).

In 2022, eastern Australia experienced one of the nation’s worst recorded flood events. Subsequently, the microbiology of flood water-immersed DFIs (n = 20) was analysed separately. *Enterococcus* spp. was the most commonly isolated pathogen, followed by *S. aureus*, *Proteus* spp., and *Acinetobacter bauumanii*. The remaining microbiology results for flood-immersed DFIs can be seen in Table 3.

*S. aureus* was the most commonly isolated microorganism regardless of the severity of infection (see Table 4). In mild infections, this was followed by normal skin flora, β-haemolytic streptococci, and unspecified gram-negative rods. In moderate-severity infections, normal skin flora was the second most commonly isolated microorganism, followed by *P. aeruginosa*. β-Haemolytic streptococci were the second most commonly isolated microorganisms in severe DFIs, followed by normal skin flora.

In chronic DFIs and in those who received antibiotics prior to admission, *S. aureus* was most frequently isolated, followed by *P. aeruginosa* and mixed anaerobes (see Table 5). Gram-positive organisms were most frequently isolated compared to gram-negative organisms regardless of prior antibiotic use (see Table 5).

## 3. Discussion

This study aimed to investigate the local causative microorganisms of DFIs in a regional centre located in a subtropical setting. A retrospective analysis of 256 microorganisms identified *S. aureus* as the predominant microorganism regardless of infection severity or acuity, which is consistent with previous research [3,11,12]. However, only 10% of the *S. aureus* samples were methicillin-resistant, which is lower than the published literature [3,13]. Based on the results of our study, locally, routine empiric methicillin-resistant *S. aureus* (MRSA) treatment may not be necessary for the treatment of DFIs.

The results of this study demonstrate a similar prevalence of *P. aeruginosa* DFIs to other (HICs) such as England (8.6%) and Italy (10.3%) [14,15]. This is in stark contrast to studies in lower-income countries, where *P. aeruginosa* accounted for 14.11% to 20.1% of pathogens [16,17,18]. Despite the subtropical climate of Northern NSW, this study did not find evidence to suggest that *P. aeruginosa* DFIs were more prevalent. The frequency of *P. aeruginosa* DFIs was similar to those seen in other HICs such as England, despite differing climates [19]. However, the rates of *P.aeruginosa* DFIs were lower than the rates found in a tropical climate despite both study sites being located in Australia [9]. The highest incidence of *P. aeruginosa* DFIs was identified in those with a history of prior antibiotic use and chronic infections (see Table 5). The pathogenesis of *P. aeruginosa* infections depends on its ability to form biofilms [20], and it has previously been associated with chronic wounds [21]. The development of *P. aeruginosa* DFIs is multifactorial. There are a myriad of factors to consider. Climate, gross national income, sanitation, hygiene, footwear use, prior outpatient antibiotic treatment failure, immunocompromised status, and the duration of the infection should all be considered when assessing an individual’s risk for developing a *P. aeruginosa* DFI [3,10,21].

The association between the severity of the DFI and the gram-stain was not statistically significant (see Table 4) despite gram-positive organisms dominating across all severity classifications. In severe DFIs, 35 (81%) gram-positive microorganisms were identified. Interestingly, *P. aeruginosa* was identified only once in the severe DFI subgroup (see Table 4). The severe *P. aeruginosa* DFI was polymicrobial, isolating Group A *Streptococcus* and MRSA, both of which are capable of causing invasive infections due to the presence of many virulence factors [22].

As expected, the microbiology of DFI differed when the DFI was immersed in flood water (see Table 3). Organisms such as *A. baumanii* and *Aeromonas hydrophila* were only identified in DFIs that were immersed in flood water. This was an expected finding given *Aeromonas* spp. can cause soft tissue infections following water exposure [23]. This reinforces the importance of following best practice guidelines for water-immersed wounds when selecting empiric antimicrobials in this patient cohort.

Of the 84 participants who had a tissue sample taken during admission, only 20 of those were taken on the day of admission. In contrast, 95 participants had a superficial wound swab during hospital admission. This is not compliant with the IWGDF/IDSA guidelines, which recommend the collection of soft tissue [5]. It is very challenging to delineate pathogen and coloniser utilising superficial wound swabs, but the decision was made to include them in the analysis due to the low number of tissue samples. The rates of major amputations (9%) found in this study were consistent with the range reported in other studies (6–21%) [24,25]. However, the rates of minor amputations (44%) were higher compared to the published literature (30%) [24].

This study found that the median duration of antibiotics in the treatment of DFI was 15 days. It was noted that there were particularly long durations with those patients who had osteomyelitis and received antibiotics following curative amputation. The median duration of antibiotics post-amputation (10 days) was significantly higher than the recommended durations of 0–5 days [4,5,26]. The post-amputation antibiotic duration did include those with positive proximal bone cultures. The study team is aware of the controversies surrounding the utility of these cultures to guide antibiotic duration [27].

Based on the results of this study, the local microbiology data support the national guidelines in Australia, according to which the majority of DFI patients should receive empiric antibiotic coverage targeted towards *S. aureus*, *Streptococci* spp., enteric gram-negative organisms, and anaerobes. Agents such as amoxicillin–clavulanate may be appropriate given the low incidence of MRSA and *P. aeruginosa* infections. However, an individual risk assessment should be undertaken for each patient prior to prescribing empiric antibiotics. During hospital admission, 126 patients (83%) received an antipseudomonal antibiotic, which was a broader-spectrum regimen than that required according to the microbiology results. Furthermore, only 27 episodes of DFI (18%) were classified as severe. Generally speaking, non-life-threatening infections should not require broad-spectrum antimicrobials initially. The local AMS program should target interventions towards lowering the inappropriate use of piperacillin–tazobactam in this setting as the overuse of antimicrobials has been shown to lead to increased costs and adverse clinical events [28]. Furthermore, the overuse of antipseudomonal antibiotics in DFIs does not lead to better patient outcomes [29].

There are multiple limitations to this study that must be acknowledged. This is a retrospective study, and the assessment of infection duration and severity relied solely on comprehensive documentation in the medical records. The study was also conducted in a small regional hospital in Australia, which may limit the generalisability of the findings. The results of this study might not be directly applicable to other sites without considering local microbiological patterns and antibiotic resistance trends. A decision was made to include superficial wound swab results to increase the sample size, but this inclusion in the microbiological analysis made it challenging to differentiate between colonisers and true pathogens. The decision to include those with positive proximal bone cultures conveys that patients with residual OM may have been included in the analysis. This may have influenced the excessive duration of antibiotics post-amputation. Finally, the number of hospital admission for flood-immersed DFIs was small, and large conclusions about the microbiology aetiology of these infections cannot be drawn. These microbiology findings also may not be directly applicable to other regions or situations without similar environmental disasters. Large-scale prospective studies comparing various climates in HICs are crucial for confirming the suggested association between warmer climates and an increased incidence of *Pseudomonas* spp. infections.

## 4. Materials and Methods

### 4.1. Study Design

An observational, retrospective analysis of 151 episodes of DFIs admitted to a regional hospital in Australia from 1 January 2021 to 31 December 2022 was performed. Individuals admitted to the hospital with a DFI were identified via the electronic medical record. All adult patients (>18 years) who had been diagnosed with diabetes and were undergoing antimicrobial treatment for DFI were included. All patients with a diabetes-related foot ulcer who did not have an infection were excluded.

### 4.2. Clinical Data

Demographic and clinical data, including the classification of infection, the acuity of infection, antimicrobial use, and surgical procedure, were obtained from the electronic medical record. Microbiology results were obtained via the local pathology database. The identification of bacterial isolates was carried out using conventional methods and followed the local microbiology laboratory standards of care.

### 4.3. Key Definitions

Prior antibiotic use was defined as any antibiotic that was prescribed in the three months prior to admission. Clinical pharmacists’ medication management plans, dispensing records, and electronic medical records were utilised to assess prior antibiotic use.

The severity of the DFI was assessed using the IWGDF/IDSA severity criteria [5]. Both the assessment of severity and acuity were completed retrospectively based on the documentation in the electronic medical record.

A deep-tissue sample was defined as any sample that was labelled as tissue within the local pathology database. A superficial wound swab was defined as any sample that was labelled as a swab within the local pathology database.

OM was assessed via medical record documentation. The treating team had to document either probe-to-bone or osteomyelitis to be assessed as osteomyelitis.

### 4.4. Statistical Analysis

Continuous data are described by the mean and the standard deviation or the median and corresponding interquartile range (IQR) as appropriate. Categorical data are expressed as numbers and percentages. A comparison of categorical variables was conducted using the χ^2^ test. *P* values were considered statistically significant at *p* < 0.05. All *p* values were 2-sided. Data were analysed using Microsoft Excel.

## 5. Conclusions

*S. aureus* was the predominant pathogen identified regardless of the DFI severity. While DFIs caused by *P. aeruginosa* were identified to be caused infrequently, a significant number of patients still received empiric antipseudomonal antibiotics. There is a need for alignment between local practices and national and international best practice guidelines. To improve DFI management, multiple areas require attention. Implementing bedside percutaneous biopsy for DFI OM and promoting tissue samples over superficial wound swabs are crucial for accurate diagnosis and targeted treatment.

The implementation of multidisciplinary team ward rounds should be considered to improve the overall management of DFIs and lower the rates of minor amputations. The local AMS program should prioritise interventions tailored to avoid unnecessary broad antimicrobials and optimise empirical antibiotic choice and duration. Optimising DFI management necessitates a multifaceted approach, including improving diagnostics, multidisciplinary collaboration, and antimicrobial stewardship. Further research and collaboration are crucial to achieving these objectives.

## Figures and Tables

**Table 1:** Patient demographics and characteristics.

Characteristic		Data
Number of episodes of care for DFI (n)		151
Age, median (IQR), years		69 (58–74)
Sex, N (%)		
	Male	104 (69)
	Female	47 (31)
BMI, median (range), kg/m^2^		29.42 (19.28–63.20)
Diabetes type, n (%)		
	Type I	11 (7)
	Type II	138 (92)
	Unspecified	2 (1)
Length of stay, median (IQR), days		7 (4–12)
Infection characteristics, n (%)		
	Acute	68 (45)
	Chronic	73 (48)
	Not assessable	10 (7)
Severity of infection, n (%) ^a^		
	Mild	20 (13)
	Moderate	101 (67)
	Severe	27 (18)
	Not assessable	3 (2)
Presence/absence of OM		
	Yes	70 (46)
	No	74 (49)
	Not assessable	7 (5)
Type of surgery ^b^		
	Minor amputation	66 (44)
	Major amputation	13 (9)
	Surgical debridement	25 (17)
Participants with tissue sample taken during admission, n (%)		
	Yes	82 (54)
	No	69 (46)
Participants with superficial wounds swabs taken during admission, n (%)		
	Yes	95 (63)
	No	56 (37)
Microbiology, n		163
	Monomicrobial, n (%)	68 (42)
	Polymicrobial, n (%)	95 (58)

Abbreviations: IQR: interquartile range, BMI: body mass index, OM: osteomyelitis. ^a^ Assessed by the research team as per the IWGDF/IDSA classification system [5]. ^b^ Major amputations were defined as lower limb amputations occurring above the ankle, whilst minor amputations were defined as lower limb amputations occurring at or below the ankle [4].

**Table 2:** The frequency of microorganisms in DFIs.

Microorganism	Acute, n (%)	Chronic, n (%)	All Superficial and Tissue Samples, n (%) ^a^
*Staphylococcus aureus*	32 (32)	37 (29)	84 (33)
Methicillin-resistant	3	3	8
Methicillin-sensitive	29	34	76
Normal skin flora	12 (12)	11 (9)	27 (11)
Coagulase-negative staphylococci	3 (3)	4 (3)	7 (3)
β-Haemolytic streptococci	8 (8)	7 (6)	19 (7)
Group A	0	1	1
Group B	4	3	8
Group G	4	3	10
Viridans group streptococci	2 (2)	Not applicable	2 (1)
Enteric gram-negative rods unspecified	7 (7)	6 (5)	17 (7)
*Klebsiella* spp.	1 (1)	2 (2)	Not applicable
*Klebsiella oxytoca*	Not applicable	Not applicable	2 (1)
*Klebsiella pneumoniae*	Not applicable	Not applicable	1 (0)
*Enterobacter cloacae complex*	2 (2)	1 (1)	3 (1)
*Enterococcus* spp.	7 (7)	10 (8)	16 (6)
Mixed anaerobes	3 (3)	10 (8)	14 (5)
*Morganella morganii*	2 (2)	3 (2)	6 (2)
*Providencia* spp.	2 (2)	1 (1)	3 (1)
*Pseudomonas aeruginosa*	2 (2)	13 (10)	17 (7)
*Serratia marcescens*	3 (3)	6 (5)	9 (4)
*Stenotrophomonas maltophilia*	Not applicable	1 (1)	2 (1)
*Escherichia coli*	Not applicable	2 (2)	2 (1)
No growth	7 (7)	3 (2)	12 (5)
*Proteus* spp.	1 (1)	2 (2)	3 (1)
Mixed enteric flora	3 (3)	6 (5)	8 (3)
*Alcaligenes faecalis*	1 (1)	Not applicable	1 (0)
*Myroides* species	1 (1)	Not applicable	1 (0)
Total	99	125	256

^a^ Includes acute, chronic, and not assessable DFIs. A chi-squared test of independence was performed to examine the relationship between *P. aeruginosa* DFIs, non-*P. aeruginosa* DFIs, and acuity of infection. The association between these variables was significant, *p* = 0.01271.

**Table 3:** The total frequency of microorganisms that were identified in DFI that were immersed in flood water (superficial and tissue) (n = 20).

Microorganism	Number (%)
*Enterococcus* spp.	3 (15)
*Acinetobacter baumannii*	2 (10)
*Proteus* spp.	2 (10)
*Staphylococcus aureus* (including MRSA)	2 (10)
Enteric gram-negative rods unspecified	1 (5)
*Morganella morganii*	1 (5)
*Pseudomonas aeruginosa*	1 (5)
*Aeromonas hydrophila*	1 (5)
*Citrobacter freundii*	1 (5)
Mixed growth of organisms	1 (5)
Normal skin flora	1 (5)
Mixed anaerobes	1 (5)
*Myroides* species	1 (5)
β-Haemolytic streptococci	1 (5)
Coagulase-negative staphylococci	1 (5)

**Table 4:** Frequency of microorganisms according to severity of infection.

Microorganism	Mild, n (%)	Moderate, n (%)	Severe, n (%)
*Staphylococcus aureus* (including MRSA)	9 (41)	55 (31)	20 (39)
Normal skin flora	4 (18)	19 (11)	4 (8)
β-Haemolytic streptococci	2 (9)	6 (3)	9 (17)
Viridans group streptococci	Not applicable	1 (1)	1 (2)
Enteric gram-negative rods unspecified	2 (9)	12 (7)	3 (6)
*Enterococcus* spp.	1 (5)	13 (7)	3 (6)
*Pseudomonas aeruginosa*	Not applicable	15 (8)	1 (2)
Coagulase-negative staphylococci	Not applicable	5 (3)	2 (4)
*Morganella morganii*	Not applicable	5 (3)	Not applicable
*Enterobacter cloacae*	Not applicable	3 (2)	Not applicable
*Serratia marscescens*	Not applicable	8 (5)	1 (2)
*Klebsiella* spp.	Not applicable	2 (1)	1 (2)
*Proteus* spp.	Not applicable	2 (1)	1 (2)
*Providencia* spp.	Not applicable	3 (2)	Not applicable
*Escherichia coli*	Not applicable	2 (1)	Not applicable
*Stenotrophomonas maltophilia*	Not applicable	1 (1)	1 (2)
*Alcaligenes faecalis*	1 (5)	Not applicable	Not applicable
Mixed enteric flora	Not applicable	9 (5)	1 (2)
*Myroides* spp.	1(5)	Not applicable	Not applicable
Mixed anaerobes	1(5)	9 (5)	3 (6)
No growth	1 (5)	9 (5)	1 (2)
Total	22	179	52

A chi-squared test of independence was performed to examine the relationship between Gram stain and severity of infection. The association between these variables was not statistically significant at *p* < 0.05.

**Table 5:** Frequency of microorganisms in DFIs according to duration of infection and prior antibiotic use.

Microorganism	Acute Infections		Chronic Infections	
	Antibiotics Prior to Admission, n (%)	No antibiotics Prior to Admission, n (%)	Antibiotics Prior to Admission, n (%)	No Antibiotics Prior to Admission, n (%)
*Staphylococcus aureus* (including MRSA)	14 (23)	18 (46)	33 (33)	4 (15)
Normal skin flora	10 (17)	2 (5)	7 (7)	4 (15)
*Pseudomonas aeruginosa*	2 (3)	Not applicable	12 (12)	1 (4)
β-Haemolytic streptococci	2 (3)	6 (15)	6 (6)	1 (4)
No growth	5 (8)	2 (5)	2 (2)	1 (4)
Mixed enteric flora	2 (3)	1 (3)	4 (4)	2 (4)
Coagulase-negative staphylococci	2 (3)	1 (3)	3 (3)	1 (4)
*Morganella morganii*	2 (3)	Not applicable	3 (3)	Not applicable
*Stenotrophomonas maltophilia*	Not applicable	Not applicable	1 (1)	Not applicable
*Proteus* spp.	1 (2)	Not applicable	2 (2)	Not applicable
*Serratia marcescens*	3 (5)	Not applicable	3 (3)	3 (12)
Enteric gram-negative rods unspecified	5 (8)	2 (5)	3 (3)	3 (12)
*Enterococcus* spp.	3 (5)	4 (10)	7 (7)	3 (11)
Mixed anaerobes	2 (3)	1 (3)	8 (8)	2 (7)
*Klebsiella* spp.	1 (2)	Not applicable	1 (1)	1 (4)
*Providencia* spp.	2 (3)	Not applicable	1 (1)	Not applicable
*Escherichia coli*	Not applicable	Not applicable	2 (2)	Not applicable
*Enterobacter cloacae*	2 (3)	Not applicable	1 (1)	Not applicable
Viridans group streptococci	2 (3)	Not applicable	Not applicable	Not applicable
*Alcaligenes faecalis*	1 (3)	Not applicable	Not applicable	Not applicable
*Myroides* species	1 (3)	Not applicable	Not applicable	Not applicable
Total	60	39	99	26

A chi-squared test of independence was performed to examine the relationship between Gram stain and prior antibiotic use. The association between these variables was statistically significant at *p* < 0.05.

## Data Availability

The original contributions presented in the study are included in the article; further inquiries can be directed to the corresponding author.
